# Supplementary material for: Organic acids and 2,4-Di-tert-butylphenol: major compounds of Weissella confusa WM36 cell-free supernatant against growth, survival and virulence of Salmonella Typhi
Source: PeerJ. 2020 Jan 20;8:e8410. doi: 10.7717/peerj.8410 (PMC6977521; doi:10.7717/peerj.8410)
Supplement: Supplemental Information 5 — Untreated, catalase-treated, and enzyme treated CFCS still kept anti-salmonella activity while NaOH-treated (acid-neutralizing) CFCS showed no inhibitory action. [file peerj-08-8410-s005.pdf]

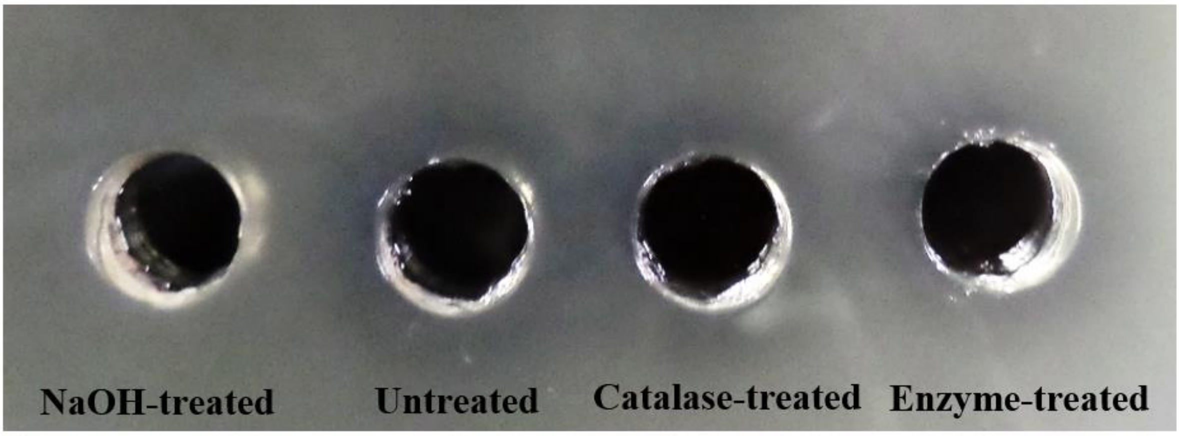

Figure S1 Inhibitory clear zone of the vicinity of observed wells by determination of antibacterial substances in treated/neutralizing CFCS.
