# Supplementary material for: Organic acids and 2,4-Di-tert-butylphenol: major compounds of Weissella confusa WM36 cell-free supernatant against growth, survival and virulence of Salmonella Typhi
Source: PeerJ. 2020 Jan 20;8:e8410. doi: 10.7717/peerj.8410 (PMC6977521; doi:10.7717/peerj.8410)
Supplement: Supplemental Information 6 — (1) lactic acid and (2) acetic acid. [file peerj-08-8410-s006.pdf]

Supplementary file

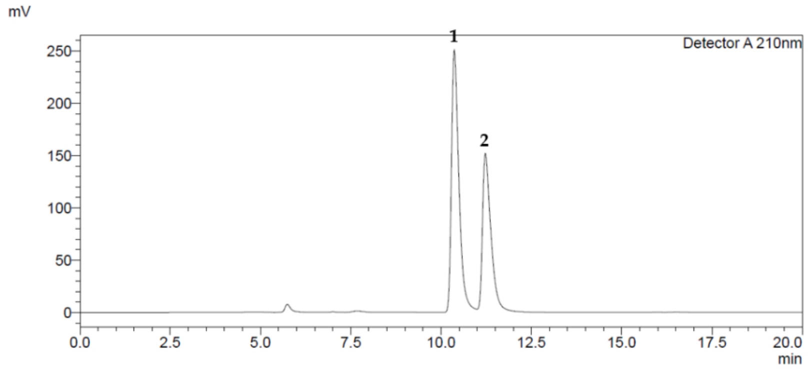

(a)

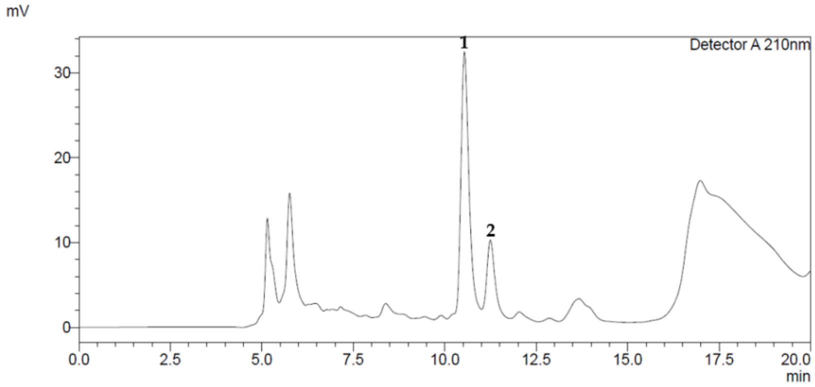

(b)

Figure S1 HPLC chromatogram of (a) standard organic acids and (b) organic acids in WM36 cell-free culture supernatant. (1) lactic acid and (2) acetic acid.
